# Using Natural Language Processing and Sentiment Analysis to Augment Traditional User-Centered Design: Development and Usability Study

**DOI:** 10.2196/16862

**Published:** 2020-08-07

**Authors:** Curtis Lee Petersen, Ryan Halter, David Kotz, Lorie Loeb, Summer Cook, Dawna Pidgeon, Brock C Christensen, John A Batsis

**Affiliations:** 1 The Dartmouth Institute for Health Policy Dartmouth Hanover, NH United States; 2 Quantitative Biomedical Sciences Program Dartmouth Hanover, NH United States; 3 Thayer School of Engineering Dartmouth Hanover, NH United States; 4 Computer Science Dartmouth Hanover, NH United States; 5 Department of Kinesiology University of New Hampshire Durham, NH United States; 6 Department of Physical Medicine and Rehabilitation Dartmouth-Hitchcock Medical Center Lebanon, NH United States; 7 Department of Epidemiology Dartmouth Hanover, NH United States; 8 Department of Molecular and Systems Biology at Dartmouth Dartmouth Hanover, NH United States; 9 The Dartmouth Institute for Health Policy Dartmouth Lebanon, NH United States; 10 Department of Medicine Geisel School of Medicine Dartmouth Hanover, NH United States; 11 Section of General Internal Medicine Dartmouth-Hitchcock Lebanon, NH United States

**Keywords:** aged adults, sarcopenia, remote sensing technology, telemedicine, mobile phone

## Abstract

**Background:**

Sarcopenia, defined as the age-associated loss of muscle mass and strength, can be effectively mitigated through resistance-based physical activity. With compliance at approximately 40% for home-based exercise prescriptions, implementing a remote sensing system would help patients and clinicians to better understand treatment progress and increase compliance. The inclusion of end users in the development of mobile apps for remote-sensing systems can ensure that they are both user friendly and facilitate compliance. With advancements in natural language processing (NLP), there is potential for these methods to be used with data collected through the user-centered design process.

**Objective:**

This study aims to develop a mobile app for a novel device through a user-centered design process with both older adults and clinicians while exploring whether data collected through this process can be used in NLP and sentiment analysis

**Methods:**

Through a user-centered design process, we conducted semistructured interviews during the development of a geriatric-friendly Bluetooth-connected resistance exercise band app. We interviewed patients and clinicians at weeks 0, 5, and 10 of the app development. Each semistructured interview consisted of heuristic evaluations, cognitive walkthroughs, and observations. We used the Bing sentiment library for a sentiment analysis of interview transcripts and then applied NLP-based latent Dirichlet allocation (LDA) topic modeling to identify differences and similarities in patient and clinician participant interviews. Sentiment was defined as the sum of positive and negative words (each word with a +1 or −1 value). To assess utility, we used quantitative assessment questionnaires—System Usability Scale (SUS) and Usefulness, Satisfaction, and Ease of use (USE). Finally, we used multivariate linear models—adjusting for age, sex, subject group (clinician vs patient), and development—to explore the association between sentiment analysis and SUS and USE outcomes.

**Results:**

The mean age of the 22 participants was 68 (SD 14) years, and 17 (77%) were female. The overall mean SUS and USE scores were 66.4 (SD 13.6) and 41.3 (SD 15.2), respectively. Both patients and clinicians provided valuable insights into the needs of older adults when designing and building an app. The mean positive-negative sentiment per sentence was 0.19 (SD 0.21) and 0.47 (SD 0.21) for patient and clinician interviews, respectively. We found a positive association with positive sentiment in an interview and SUS score (ß=1.38; 95% CI 0.37 to 2.39; *P*=.01). There was no significant association between sentiment and the USE score. The LDA analysis found no overlap between patients and clinicians in the 8 identified topics.

**Conclusions:**

Involving patients and clinicians allowed us to design and build an app that is user friendly for older adults while supporting compliance. This is the first analysis using NLP and usability questionnaires in the quantification of user-centered design of technology for older adults.

## Introduction

### Sarcopenia

Sarcopenia is the loss of muscle mass, strength, and function, which occurs with aging and is associated with serious health consequences such as disability [1], morbidity [2], and mortality [3]. Although the etiology is complex, mitigating its development is important in preserving long-term muscle function. Recommended treatments include resistance exercise programs [4] and protein supplementation, such as amino acids including creatine [5] or leucine [6], and vitamin D [7]. Strengthening exercises normally prescribed by physical therapists are cost-effective, safe, improve physical functioning [8], and prevent muscle loss [9]. Exercises to enhance muscle mass and strength conducted 2 to 3 times per week normally consist of using resistance exercise bands or weights—materials that are easily available and can be used in medical, community, or home-based settings.

### Building Usable Solutions

Although the evidence base is clear for the efficacy of such treatment strategies, an estimated 40% of patients fail to adhere to their recommended regimens [10,11], making it difficult for clinicians to understand a patient’s progression through therapy. Observation of activity provides a gold standard for therapists to tailor, evaluate, and encourage treatment regimens. However, direct observation is not practical or feasible in health systems and specifically for older adults facing barriers of travel and transportation to in-person visits [12]. Remote medical sensing and mobile health (mHealth) technologies have the potential to help patients and providers understand and track adherence and progression through therapy, and overcome some of the major barriers to attending in-person sessions. Technology has been used to track and communicate with patients with chronic diseases [13] and has demonstrated the ability to improve compliance with treatments [14].

As older adults have specific sensory needs and different perceptions of mHealth [15], it is important to employ user-centered design methods to ensure that the final device and app meet the needs and preferences of older adults [16]. A *user-centered design* incorporates the end user of a product in all phases of the design, ensuring that the result aligns with the users’ needs. Qualitative methods of user-centered design incorporate interviews and the assessment of constructs identified through these conversations. Such methods are labor intensive and require specific training. Some of these barriers can be surmounted using quantitative techniques such as *natural language processing (NLP)*, which requires relatively little computational resources and leverages existing workflows and software pipelines. NLP has many applications, such as information retrieval; in the medical field, it is increasingly used to extract topics in electronic health record data [17]. In this context, *sentiment analysis* has been used to examine the perception of health care, drugs, treatment, or illnesses using social media data [18], although it has started to be used in a broad range of applications, from analyzing investor earnings calls [19] to interactions with chatbots [20]. These methods could be used beyond determining if people enjoy an investor call, health care system, or chatbot to assess how a person perceives a health product in development before a device or program has been finalized. Using them in the design and development of mHealth products for specific patient populations could lead to more rapid and accurate determination of how they feel about an mHealth product and how it could be improved without the burden of questionnaires.

We previously developed a Bluetooth-connected resistance exercise band that had the potential to provide feedback to both patients and providers on exercise compliance and treatment progress [21,22]. The addition of a mobile app permits real-time monitoring and has the ability to use cloud-computing resources to provide feedback, force, and detection of exercise repetitions to clinical or research teams. For patients with sarcopenia, connecting a resistance band to an app provides a platform for them to understand their progress through each exercise as they proceed through the regimen. With a suitable *dashboard*, the app allows clinicians to monitor not only compliance for each patient but also their entire patient population. As much of the user-centered design process is dependent on both interviewing users to assess their perception and quantifying it through questionnaires, we also sought to determine if the data generated through these conventional methods could also be analyzed through NLP and sentiment quantification methods. This exploratory work leverages the interviews and conversations that were collected during user development, quantifying them using new methods. A preliminary examination of correlation with questionnaires can also help shed light on how they may be interpreted in the future. To our knowledge, this is the first study to apply NLP and sentiment analysis to interviews in a usability study for older adults.

### Objectives

This study aims to create a mobile app for older adults to monitor their use of a Bluetooth-connected resistance band and to examine whether data collected through this process could be used for NLP and sentiment analysis.

## Methods

### Study Population

Participants were recruited through a primary care clinic at Dartmouth-Hitchcock in Lebanon, New Hampshire, a rural health care institution caring for 1.5 million patients in New Hampshire and Vermont. Word-of-mouth and study posters provided the main source of referrals. All study activities were conducted at the community-based Dartmouth-Hitchcock Aging Resource Center. Eligible participants were English-speaking, community-dwelling (eg, not residing in a nursing home or assisted living center) older adults aged 65 years or older without a self-reported diagnosis of dementia. There were no other specific inclusion or exclusion criteria. Clinicians were faculty members of the Section of General Internal Medicine at Dartmouth-Hitchcock. All participants were provided a research information sheet before the start of the study and were recruited as a convenience sample from Dartmouth-Hitchcock. The study was approved by the Committee for the Protection of Human Subjects at Dartmouth College and the Dartmouth-Hitchcock Institutional Review Board.

### App and User-Centered Design Process

We built an app for Android, optimized for the Samsung Galaxy Tab A tablet. We chose this particular tablet for its large screen, Android operating system, and relatively low price; all factors were previously identified by patients. The app, written in JavaScript, allowed the user to sign up, connect to a Bluetooth-enabled resistance band, watch a video of an exercise, and record the data from the resistance band. Each exercise video consisted of an individual completing a specific exercise while verbally explaining it. The first exercise video was created without input from patients to provide an initial example of an exercise video.

The user-centered design process consisted of 3 rounds. Interviews with individual patients and clinicians were conducted in all rounds of the study by the same 1 or 2 research assistants. Participants were asked open-ended questions about their use of technology, their preferences on iterations of the app, and their reactions to design images and content, all of which were documented by the interviewers as field notes. Field notes consisted of observations of the user discussing and using the app. After each round, the developers, designers, research assistants, and researchers would meet to review interview notes, discuss interviewees’ perceptions, and determine issues discovered in the round that should be addressed. The team then determined potential solutions, deciding which were most feasible, and updated the design and content. Individuals only participated in a single round of development. We recorded the interviews and later used a commercial transcription service to produce a transcript for each interview.

Round 1 (predevelopment) consisted of 6 older adults. We evaluated their general perception of mHealth needs, physical activity, the Bluetooth-connected resistance exercise band, and the initial exercise video. As this round occurred before app development, the study team did not ask participants for feedback on a specific app; as such, they were not given System Usability Scale (SUS) and Usefulness, Satisfaction, and Ease of use (USE) questionnaires. Interviewees were asked about their exercise habits, their technology habits, their use of technology with exercise, and how they think they may use technology with exercise. Information from round 1 allowed the app developers to construct a prototype app for round 2 using initial designs with black and white mockups of the app.

**Figure 1:**
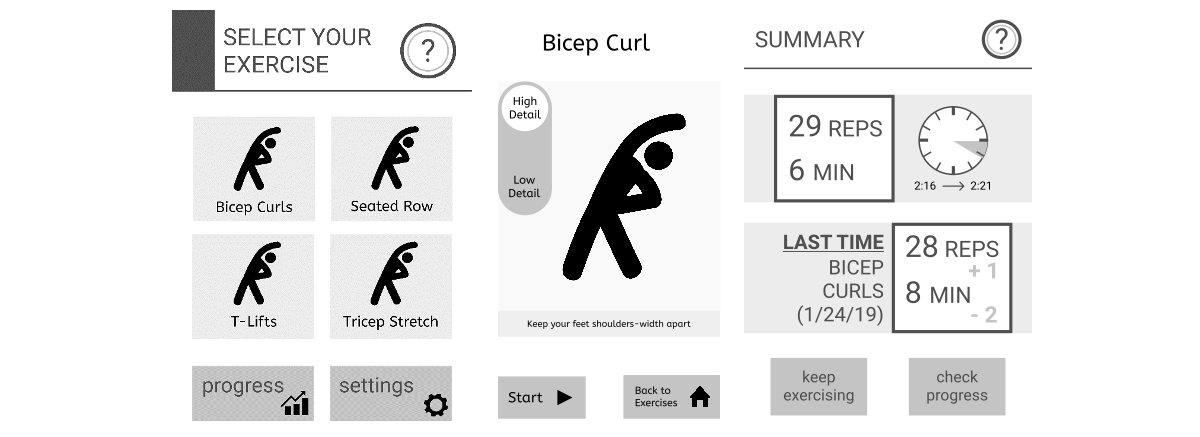
App versions for round 2 (round 1 did not use wireframes) consisted of black and white wireframes of the exercise selection screen and a preworkout screen.

**Figure 2:**
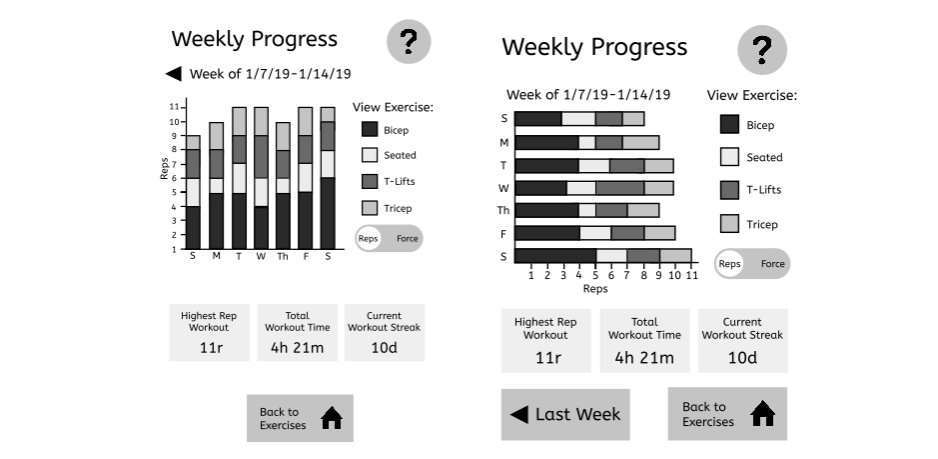
The App version for round 2 also had two different versions of the weekly progress screen shown to participants in random order.

During round 2 individual interviews, the team presented the updated exercise video to 3 clinicians and 4 patient participants and asked them to provide oral feedback. We then showed black and white wireframes of the app to the participants (Figure 1). Two versions of a weekly progress summary screen were shown in random order. Version 1 had toggles to switch between viewable summary data with a back arrow in the upper left-hand corner and vertical bar chart of repetition counts. Version 2 had buttons to switch between viewable summary data with a back button at the bottom of the workout summary screen and a horizontal bar chart of repetition counts (Figure 2). We also showed participants 2 approaches for displaying the number of completed repetitions in a workout: a vertical bar chart and a horizontal bar chart (Figure 2). Interviewers used think-aloud [23] and verbal prompting methods to encourage interviewees to share their thoughts about the function of each button and chart and their designs.

**Figure 3:**
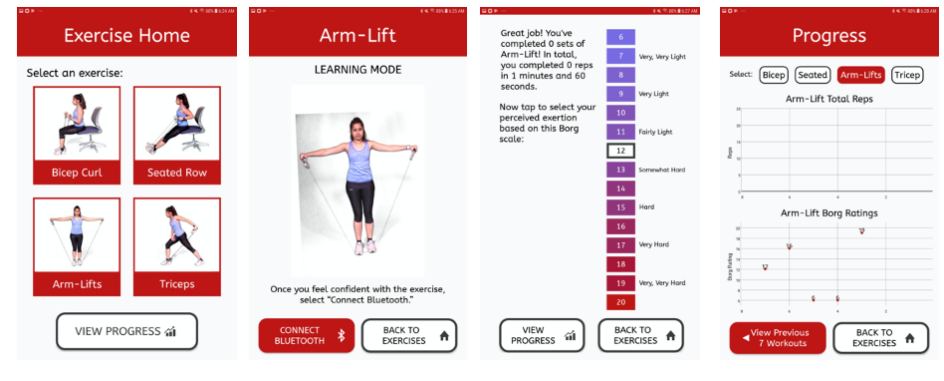
The app versions for round 3 screens were in color, had the embedded video with a low-contrast background. Here, we present the home screen, the preworkout screen, the workout screen, the Borg Scale of Perceived Exertion questionnaire, and the progress screen.

Round 3 included 3 clinicians and 6 patients. This round consisted of asking participants to start an exercise video and navigate through an exercise summary of videos in the prototype app. Each exercise video consisted of a person performing the exercise while describing how to position and move their bodies to complete the exercise successfully. Interviewers again used think-aloud and verbal prompting methods when asking the interviewees to explore the app and start a workout (Figure 3).

### Usability and Acceptability Questionnaires

After each round 2 and round 3 interview, the study team asked participants to complete 2 validated questionnaires: the SUS [24] and the USE scale [25,26]. The SUS was developed for the global assessment of a system’s usability and consisted of 10 questions, each on a 0- to 4-point Likert scale, with an overall score range of 0 to 100, following a scoring algorithm. The USE scale was developed for broad application in technology development in private industry and comprises 30 questions, each on a 1- to 7-point Likert scale and is broken down into 4 sections (usefulness, ease of use, ease of learning, and satisfaction). Each interviewee filled out both questionnaires after the interview session.

### NLP and Sentiment Analysis

The study team subsequently used NLP methods to process the deidentified interview transcripts after all the rounds and interviews were completed. We separated utterances by speaker, removing interviewer data. We removed stop words and calculated the term frequency-inverse document frequency at the interviewee level. We then used latent Dirichlet allocation (LDA) [27] topic modeling to examine the content of the core concepts discussed in each interview. An LDA analysis is a generative probabilistic model that groups words based on how related they are—these clusters of related words are termed *topics*. LDA assumes that text is a mixture of topics that have a probability of occurring in each sentence and interview. LDA methods have been described in depth in several recent medical informatic manuscripts [28-30]. To examine the feature space of topic clusters from 2 to 60 using 4 different metrics [31-35] to calculate ideal cluster numbers, we used the mean number of ideal clusters (k=8) in the LDA analysis. We reviewed the 15 words most associated with each topic cluster and named them based on the concept that the authors believed they represented most accurately. This iterative process involved discussing how words could be used differently for an underlying concept or topic. Names were then decided on by consensus.

We used the Bing sentiment lexicon to define positive and negative words in our sentiment analysis [36]. The positive and negative sentiment words within each interview were then added, where positive sentiments had positive values (+1) and negative sentiments had negative values (−1). All other words had no value (0). The final sentiment value consisted of all the mean sentiments; thus, greater positive numbers indicate greater positive sentiment, whereas lower negative numbers indicate greater negative sentiment.

### Statistical Analysis

We presented descriptive characteristics as mean (SD) and count (percentage). We determined differences in questionnaire values using the student *t* test. We used univariate and multivariate linear regression models to test the association between the total sentiment of the interview per individual (covariate of interest) and usability and acceptability questionnaire scores. Outcomes were the SUS score and each component of the USE questionnaire. All qualitative interview data were transcribed, managed, and coded in *Dedoose*, completed after categorizing data excerpts at each stage. Field notes were also obtained and aggregated. All codes were reviewed, and we identified positive or negative themes among the participants and juxtaposed them with the sentiment analysis. Multivariate linear models included age, sex, and subject group (clinicians vs patients). We conducted all analyses in R version 3.6.0 [37], and significance was defined as <.05.

## Results

### Study Population

We recruited 22 participants (6 clinicians and 16 patients) to review the app design and functionality. The age of the clinicians was significantly lower than that of patients with a mean age of 49 (SD 9) years; the mean age of the patients was 76 (SD 5) years (*P*<.001). The majority of the participants were female (17/22, 77%), and all were of non-Hispanic white ethnicity (Table 1).

**Table 1:** Study participant characteristics (N=22).

Participants		Clinicians (n=6)		Patients (n=16)	
**Age (years)**					
	Mean (SD)		49.0 (9.4)		75.56 (5.2)
	Minimum to maximum		37 to 51		66 to 85
Male, n (%)		2 (33.3)		3 (18.8)	
Non-Hispanic White, n (%)		6 (100)		16 (100)	

### Mobile App Design and Development

In round 1 (predevelopment), 6 patient participants were asked about their exercise behavior, how they used mobile devices, and their perception of the initial exercise video. All participants had experience with smartphones and computers and expressed that they were interested in using technology in a physical therapy program. Participants found it difficult to follow the exercise video with a low-contrast background and without each specific movement shown in close-up detail. They also found it difficult to hear the audio of the video or to distinguish the words being said. Participants expressed that they needed help in counting repetitions, knowing the exercise that they needed to do, and having clearly labeled buttons, as older adults may not be able to assume functionality as well as younger people. This led to designs that contained labeled buttons, such as back and home, along with some repetition counting functionality. Patients also hoped that technology can help provide feedback and guidance and thus impart confidence in completing exercises while at home. Finally, patients preferred the use of tablets over phones because of their larger screen size as such designs were based on tablet screen layouts and not smartphones.

Round 2 focused on basic functionality using black and white wireframes. In total, 4 patients and 3 clinicians indicated that a vertical stacked bar chart was more interpretable than horizontal bar charts when reviewing the progress of previous exercise data. Patients found it difficult to follow the new video and considered the video instructions to be too fast and not detailed enough to understand the physical positioning required to complete the exercise. In addition, patients wanted to have large font text instructions along with the option to change the audio frequency of the video to help those with hearing impairment who may have difficulty hearing higher pitches. Clinicians had no issues with the videos and commented more about the content of the text on the screen. Specifically, they suggested changing the word associated with the videos from *recording* to *video* as one might think it will take a recording instead of playing one. Clinicians also suggested that we added the Borg Scale of Perceived Exertion as a measure of relative difficulty.

Round 3 involved 3 clinicians and 6 patient participants. Patients and clinicians reviewed the final videos and were asked to navigate through the app on the tablet in independent interviews. Some indicated that it would be helpful to include a separate video that taught the user how to complete the exercise and described in detail how the exercise was executed. This could be used along with the normal video that went through the exercise in real time to guide the user through it. Others indicated that some patients may have trouble with the technology and recommended that the app provide a means for the patient to call and talk with a live person.

### Usability and Acceptability Questionnaires

All recruited clinicians and patients completed the usability and acceptability questionnaires. There was no statistically significant difference between patients and clinicians in their perceived use of, or satisfaction with, the app measured through either the SUS (mean 66.8, SD 16.5 vs mean 65.8, SD 7.7; *P*=.90) or the USE questionnaires (usefulness: mean 37.2, SD 18.0 vs mean 48.0, SD 5.2; *P*=.18; ease of use: mean 54.1, SD 16.4 vs mean 66.0, SD 9.1; *P*=.13; ease of learning: mean 22.4, SD 6.3 vs mean 27.5, SD 0.8; *P*=.07; and satisfaction: mean 33.3, SD 14.1 vs mean 44.3, SD 3.6; *P*=.08).

### NLP and Sentiment Analysis

**Figure 4:**
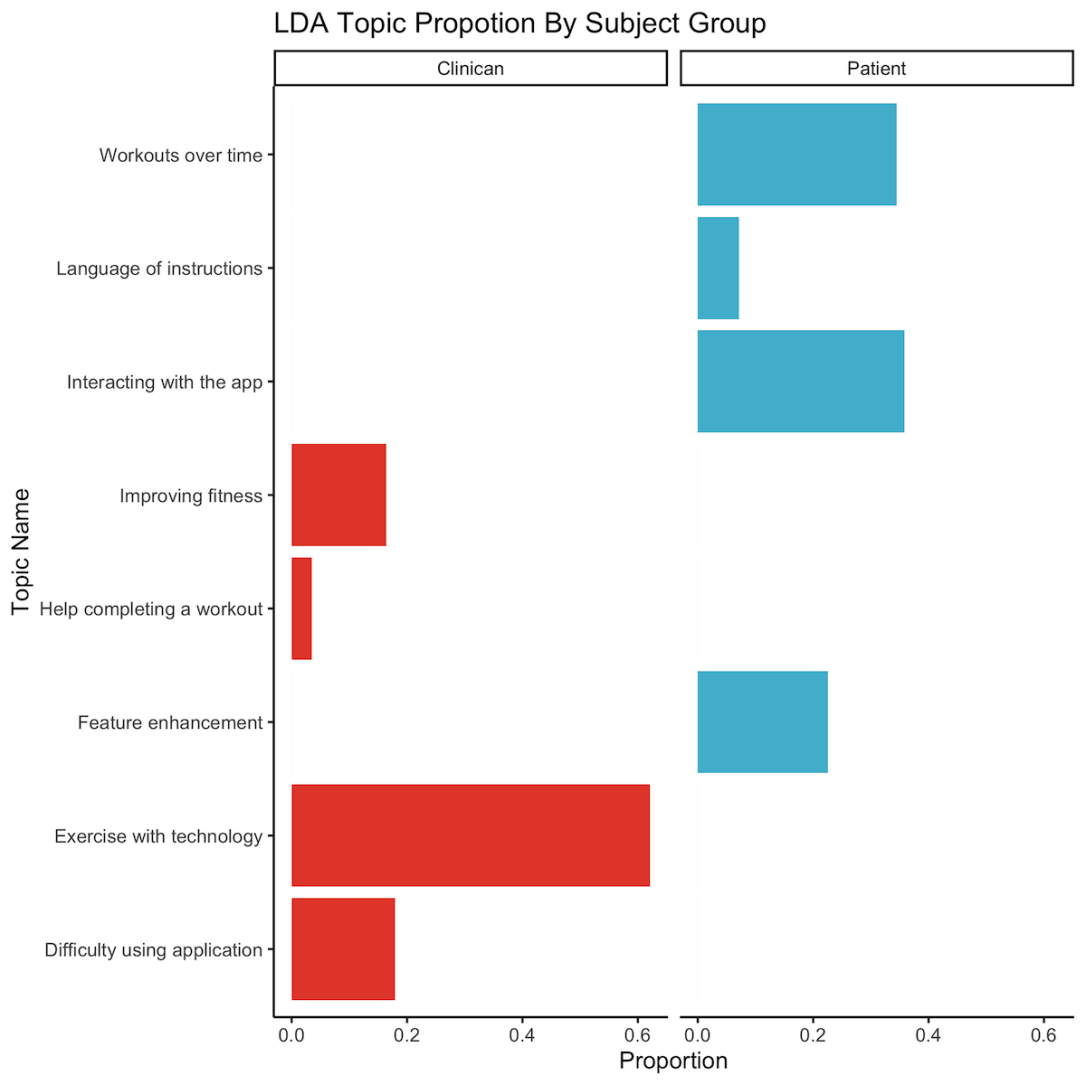
LDA identified interview topics for clinicians and patients from transcripts. Each topic was named by examining the words within each topic and qualitatively determined the concept that summarized them. Here we display the proportion of the interview that was identified as each topic for both clinicians and patients. LDA: latent Dirichlet allocation.

**Figure 5:**
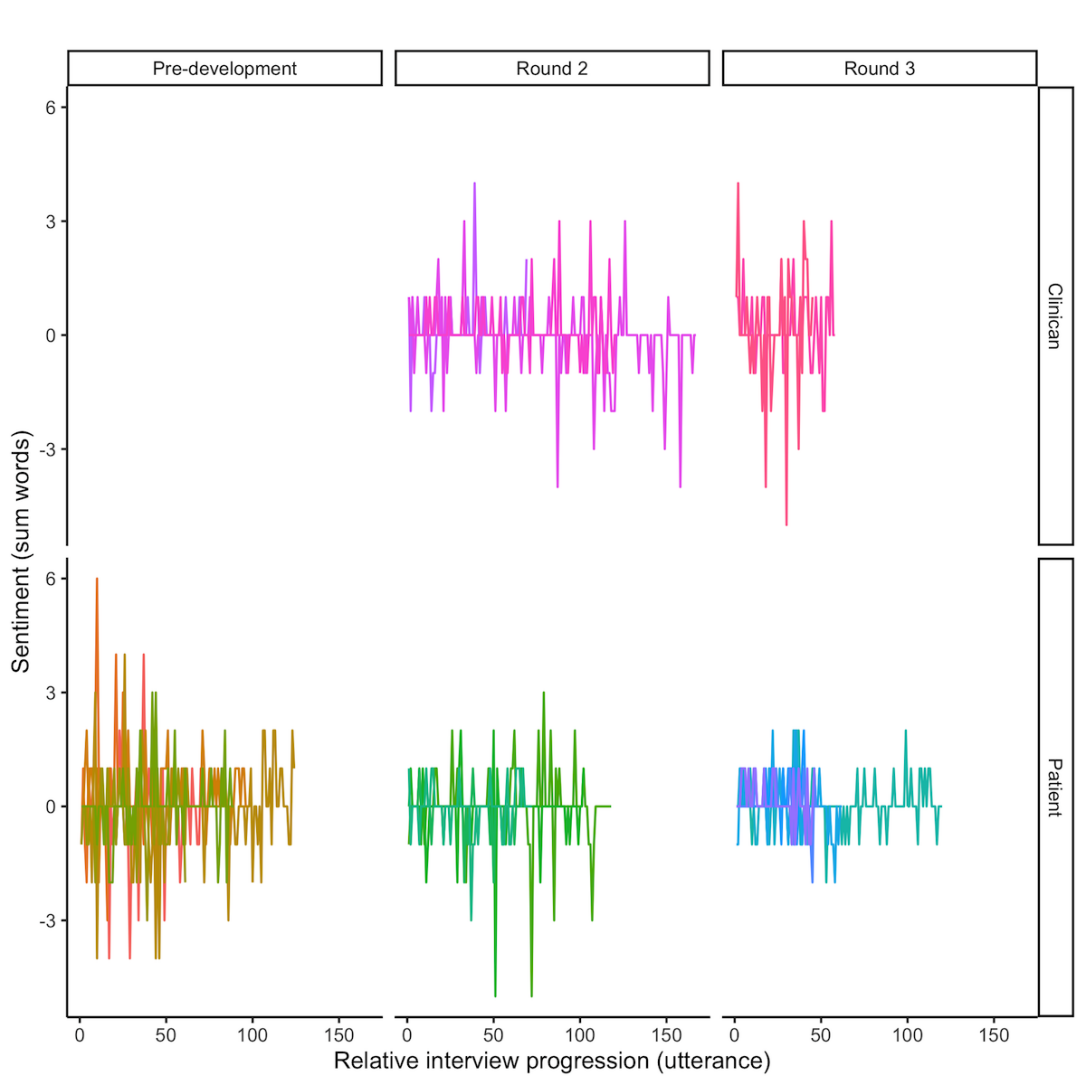
Sentiment over the course of the interview for clinicians and patients in each round of interviews. Negative sentiment values represent negative sentiments, while positive sentiment values represent positive sentiments.

LDA identified 8 core topics from the transcribed interview corpus. The distribution of these topics was different between clinicians and patients (Figure 4). Clinicians’ core topics included workouts over time, language of instructions, interaction with the app, and feature enhancement, whereas the core patient topics were improving fitness, help completing a workout, exercising with technology, and difficulty using the app. The total mean sentiment of each round increased from −0.17 (SD 15.92) in the predevelopment round 1 to 1.57 (SD 10.20) in round 2 and to 6.00 (SD 3.85) in round 3. Across all interviews, the maximum sentiment for a single statement was 6, whereas the minimum was −5 (Figure 5). The overall mean sentiment per phrase was 0.47 (SD 0.21) and 0.19 (SD 0.21) for clinician and participant interviews, respectively. When adjusting for age, sex, and subject group, the total sentiment value was significantly associated with the SUS, with each additional net positive statement associated with a 1.36 times increase in the SUS score (*P*=.01; Table 2). These results were not observed in any of the components of the USE questionnaire when adjusted for the same covariates (not shown). Table 3 presents representative statements that our sentiment analysis found positive and negative for both patients and clinicians.

**Table 2:** Univariate and adjusted model for the System Usability Scale score with sentiment.

Characteristics		Univariate beta (95% CI)	*P* value	Adjusted beta (95% CI)	*P* value
Sentiment		1.11 (0.26 to 1.97)	.01	1.38 (0.37 to 2.39)	.01
Age (years)		0.03 (−0.51 to 0.58)	.89	0.59 (−0.72 to 1.96)	.25
**Sex**					
	Female	Reference	N/A^a^	N/A	N/A
	Male	−3.81 (−21.58 to 13.97)	.65	−2.59 (−18.45 to 13.26)	.59
**Subject group**					
	Clinicians	Reference	N/A	N/A	N/A
	Patients	−1.25 (−18.04 to 15.54)	.87	−8.48 (−42.74 to 25.89)	.72

^a^N/A: not applicable.

**Table 3:** Representative statements by patients and clinicians that were found to be positive or negative by sentiment analysis.

Participants, Statement types		Representative statements
**Patient**		
	Positive	“Oh, that’s wonderful for the upper arms, in and out. That’s wonderful. Yeah. I like that one.” “Honestly, it makes sense to be at the bottom. But I think most programs are at the top. I like the top.” “Technology. Well, I used to be quite good when I was working, because I had to keep up with the technology… [B]ut I use [MS] Word, I use email, and I text. I mean I’m not a real techie and I wouldn’t say I’m up to date on all the latest gizmos that it’s possible to do on my laptop or my smartphone. What I try to keep up, I can do more than is required of the life that I’m living.”
	Negative	“Okay. Restraint, I missed a little bit. I had trouble with the palms up for a minute. Elbows straight I missed altogether, and slow return.” “Slow, slow. The key word with us elders is say it slow, slowly, key, and phone numbers too.” “You gotta go louder, I can’t hear much. I’ve got my hearing aids on, but they’re useless.” “Golly. The only thing that ever frustrates me is if something goes wrong technically and I don’t know what to do about it. And usually it’s something very simple. If I think about it, I very often can fix it myself. But I have a tech guy who comes in and helps me every once in a while.”
**Clinician**		
	Positive	“I think overall the usability should be good, I think just these little tweaks here and there to make it just so they can ... I think in general it’s fairly easy and at least for, you know, does show some basic ... you don’t have to be super computer literate.” “No, I know what you guys are doing, it’s cool. Okay, so it’s really easy to discover the Bluetooth device; which is awesome. And same deal, back to ... It’s really simple, it has a two layer architecture; which is really, really simple. Which I really like, actually.”
	Negative	“[My] mindset is that I want it to go this pathway in order to start my freaking exercises, record them, and I want to press the minimal buttons to get to where I want to go. That’s what anyone would want to do, and it’s misleading with that video, because it makes me feel like, oh, now I can start my exercise by pressing play, but no, I’m not starting my exercise.” “Now, oh, I have to think. I have a working brain, but many people are slower, and they’re tired, and this is just a lot of crap going on. I’m not happy with this. But yeah, I press something, and nothing happened, which is also annoying.”

## Discussion

### Principal Findings

Through user-centered design practices, we developed a mobile app for a Bluetooth-connected resistance exercise band. For each round of development, the number of positive sentiments increased, although there was no significant difference in either patients’ or clinicians’ sentiment of the app in interviews. In the adjusted models, we demonstrated that increasing the total sentiment of an interview was positively associated with SUS scores. Finally, there are meaningful differences in the topics that were identified in the clinician and patient interviews through both qualitative and quantitative methods. Our exploratory results also suggest that NLP and sentiment analysis could be considered in future user-centered design processes.

Our experience with user-centered design aligns with previous insights involving older adults in the development of exercise games [38], active and assisted living [39] and wearable technologies [40]. Our results expand on these prior studies, as we found that patients and clinicians had different perspectives. Our approach allows additional methods for triangulation that are observed in formal mixed methods designs [41]. These results highlight the importance of seeking end user input and involving both clinicians and patients in the design of medical technology as they demonstrate measurable differences in their viewpoints.

The use of sentiment analysis in clinical research has typically been used to assess social media‑based opinions around a specific topic such as hospital quality [42], palliative care [43], or tobacco use [44]. The methods used in the sentiment analysis vary—some build and train a model based on the study data, whereas others use open-source or private algorithms [18]. We used an open-source library, which allows others to use the same methodology. With preliminary correlation with the USE scale, the sentiment analysis suggests that it may assess similar domains in this context. Owing to the association between sentiment analysis and usability scores, we believe that a sentiment analysis can be actionable within the development cycle without the burden of a questionnaire. This can be accomplished with automated analytic scripts that parse, clean, and analyze interviews after they have been completed. However, future validation studies, similar to factor analyses for survey methods, should be considered.

We sought to increase external validity and reproducibility by using the public Bing sentiment library [45] because of its previous use and implementation simplicity. This lexicon enabled us to measure variations within and across interview texts, as in other sentiment analyses [18]. LDA methods assume that each text grouping is a mixture of topics and that such topics are a probabilistic mixture of words. Previous studies have applied these methods to clinical notes to identify topics predictive of specific diseases such as dementia [46] and heart failure [47]. In theory, these methods could be used to predict such outcomes (through notes alone) before a diagnosis. The concept that a conversation or interview is also a mixture of topics that comprise probabilistic mixtures of words makes sense as the interviewee will express different topics that are important to them. Here, we did not use LDA-identified topics in prediction but in the identification of concepts that could assist in improving development and as a proof-of-concept that there are differences between the patients’ and clinicians’ perceptions of remote medical sensing. Analogs to naming discovered themes in the qualitative analysis (here the identified latent topic) must be entitled by the researchers themselves through interpretation, as it is not an automated process. Discovering the clear differences in topics between patient and clinician interviews validates the need to include both stakeholders in the design and development of remote medical sensing apps. This is an extension of the coproduction concept wherein patients and clinicians combine to help support patients contributing to the management of their own conditions [48,49]. As the use of sentiment analysis and LDA evolve, our results suggest that they can potentially be used alongside more traditional qualitative methods to examine the concepts and perceptions within the context of user-centered design and interviews.

### Strengths

This study has several strengths. First, we were able to recruit both older adults and clinicians to participate in a user-centered design. Such an approach provides differing perspectives in addressing complex usability problems. Second, we demonstrated that researchers can use a sentiment analysis and LDA-based topic mapping for interviews with older adults and clinicians. Our evaluation goes beyond traditional qualitative methods that may not necessarily capture sentiments of developing specific apps. Finally, this study paired these evolving quantitative methods with known usability questionnaires to demonstrate the relationship between overall interview sentiment and perceived utility.

### Limitations

Although our pilot study sample size was not very large, we were able to reach a total number of participants that allowed us to isolate conceptual themes within the suggested ranges for theoretical saturation [50]. The small sample size, magnitude, and significance of the regression results are less important; however, our goal was to demonstrate an important direction of effect for future research. The scope of the study is also limited to the development of the mobile app; we did not examine the efficacy of its implementation within a patient population of older adults or clinicians. We used a single sentiment library rather than building a sentiment library that worked for this data set. Our approach should be further tested in other medical usability studies to verify these results. As all NLP and sentiment analyses were conducted after development, we did not examine how they could be used in the active development cycle and could potentially be considered in future design processes. As such, we did not have an *a priori* hypothesis of effect size, and this analysis serves as formative work for others interested in using NLP and sentiment analysis. In addition, LDA-identified topic clusters may be difficult to interpret; here, we qualitatively examined the words most associated with the topic and named them for their underlying concept. Finally, using sentiment analysis around spoken words in the medical and health setting might not be as accurate as its use in a written corpus [51].

### Future Work

With the development of the mobile app, we will next focus on deploying it and the resistance exercise band in real at-home experiments. Although challenges still exist in mHealth’s ability to impact health behavior [52], monitoring adherence and engagement is the first step. Not all of the insights identified by patients and clinicians could be implemented in this short study and should be added in future versions. Furthermore, new studies will examine the efficacy of the use of the app and its connected device.

The utility of NLP-based methods and sentiment analysis should be further examined in the context of interviews and usability questionnaires within the medical field with larger samples. Although we demonstrated correlation with a usability questionnaire, more work needs to be done to understand how sentiment analysis could be used in the context of conversations and how they may replace or augment questionnaire-based methods. These methods could be used within the development of technology used by patients and providers to assess how they are perceived. The use of NLP and sentiment analysis pipelines could be set up before interviews are conducted, allowing for analysis to occur immediately after the interview. Although not possible in this study, insights from NLP and sentiment analysis could then be incorporated into the design. Finally, additional research needs to examine how interviews could be structured to most effectively use NLP and sentiment analysis. Specifically, asking participants to describe how they felt during different uses or aspects of use may generate data that are actionable within the user design cycle. For user-centered design, many questionnaires are developed for assessing a fully functional product and not one that is partially functioning or disparate parts of a product, making it difficult to use such questionnaires in a pre-post fashion. Using sentiment analysis may allow researchers to more reliably compare the quantification of users’ thoughts through the developmental rounds.

### Conclusions

User-centered design with both patients and clinicians allowed us to build an app that older adults can use. This is the first analysis that used NLP and usability questionnaires to quantify the user-centered design of technology for older adults.

## Abbreviations

LDA: latent Dirichlet allocation
mHealth: mobile health
NLP: natural language processing
SUS: System Usability Scale
USE: Usefulness, Satisfaction, and Ease of use scale
